# The Concept of Harm and the Significance of Normality

**DOI:** 10.1111/j.1468-5930.2012.00574.x

**Published:** 2012-09-18

**Authors:** Guy Kahane, Julian Savulescu

**Affiliations:** Oxford Uehiro Centre for Practical Ethics, University of OxfordOxford, OX1 1PT, UKguy.kahane@philosophy.ox.ac.uk; Oxford Uehiro Centre for Practical Ethics, University of OxfordOxford OX1 1PT, UKjulian.savulescu@philosophy.ox.ac.uk

## Abstract

Many believe that severe intellectual impairment, blindness or dying young amount to serious harm and disadvantage. It is also increasingly denied that it matters, from a moral point of view, whether something is biologically normal to humans. We show that these two claims are in serious tension. It is hard explain how, if we do not ascribe some deep moral significance to human nature or biological normality, we could distinguish severe intellectual impairment or blindness from the vast list of seemingly innocent ways in which we fail to have as much wellbeing as we could, such not having super-intelligence, or not living to 130. We consider a range of attempts to draw this intuitive normative distinction without appealing to normality. These, we argue, all fail. But this doesn't mean that we cannot draw this distinction or that we must, implausibly, conclude that biological normality does possess an inherent moral importance. We argue that, despite appearances, it is not biological normality but rather statistical normality that, although lacking any intrinsic moral significance, nevertheless makes an important moral difference in ways that explain and largely justify the intuitive distinction.

## 1. An Intuitive Moral Distinction Concerning Harm

It is very widely held that:

(1) to be severely intellectually impaired, paraplegic, blind, or to die in one's 20s is to suffer, in different ways and degrees, from serious disadvantage and harm.^1^

Most of us take blindness, for example, to be a very significant harm. This is why we feel sympathy when we hear about someone who lost their sight, and why so much effort is put into finding ways to preserve or restore sight. We similarly feel deep regret when we hear about a teenager who died in an accident — not just because of the loss of life, but also because they died so *young*. We certainly do not feel as much regret when we hear of the passing away of a 100-year-old.

Although harm is undeniably a central moral notion, it is not yet well understood. It is widely agreed that the harmful is what reduces wellbeing, and makes a life go worse.^2^ But these are ambiguous claims. They can be understood temporally: something is harmful if it causes someone to have less wellbeing then they had before — think of someone who before an accident could see, and is now blind. But this understanding of harm faces familiar problems. It cannot explain how blindness might be harmful to someone who was born blind, or became blind as a foetus. In these cases, the blindness did not cause a reduction of a prior level of wellbeing. This account also cannot explain why it is harmful to prevent someone's greater future flourishing. If a safe cure to a life-long disease is maliciously withheld from a patient, this does not cause any *reduction* in the patient's wellbeing, and therefore, absurdly, won't count as harm on this view. For these reasons, it is more common to understand harm counterfactually. On this view, a harmful action, event or condition is what makes a life worse than it *would have been otherwise*, if that action or event had not occurred, or if that condition was not present.^3^ The idea is that the life of someone who was born unable to see is worse than the life they would have had, if they *could* see. In this sense, it is a harm for that person to be blind.^4^

But this account of harm immediately faces a serious problem. For consider the following list:

(2) to have less than an IQ of 160, to lack great artistic talent, or to live less than 130 years.

This list *also* describes conditions which, compared to the counterfactual alternative, arguably make a life go worse.^5^ Yet no one would describe the items on (2) as instances of serious disadvantage, harm or misfortune; someone who complained that they do not have the IQ or artistic talent of a genius would get little sympathy from us. Indeed, we could conceive of numerous further ways, some entirely farfetched, in which our current limitations reduce our wellbeing compared to alternatives — perhaps our lives could be better if we had telepathic powers, or could fly, and so forth. Again, it seems absurd to describe these limitations (and the conditions that underlie them) as serious harms. It seems to render the notion of harm empty of content to claim that we are harmed by not having X-ray vision.

There seems, then, to be a deep normative distinction between the items on list (1) and those on list (2). What could ground this difference?

It is natural to think that what makes only the items on (1) harmful is that they deviate, in a negative way, from what is natural to humans — from normal human species functioning and capacities. It is natural for humans to see, walk and have certain cognitive capacities, and, perhaps, to live to a certain age. None of this is true of having an IQ of 160 or living to 130, let alone of the fact that we lack various science fiction or supernatural capacities.

It is natural to appeal in this way to human nature to explain the intuitive normative distinction between (1) and (2). Indeed the idea that our biology sets the standard for human flourishing remains attractive to some philosophers.^6^ But we believe that this answer won't do. Deviation from the biologically normal cannot explain this normative distinction because, in itself, biological normality has no moral significance. To the extent that modern biology even provides us with a useful notion of human nature, it is of a set of characteristics that was selected by a blind evolutionary process. If nature draws a line, it is an entirely arbitrary line, with no moral authority.^7^

Moreover, given the average lifespan of hunter-gatherers, it's hardly plausible to hold that it is natural for humans to live to 70, but unnatural for them to live to 130. But to appeal to the fact that it is *statistically* normal for modern humans to live beyond their twenties, and not much further than their seventies, is to appeal to what is even *more clearly* a merely arbitrary distinction. Why should it morally matter where one happens to fall on the contingent current bell curve of normality? A striking example of this arbitrariness is provided by the common definition of intellectual disability as an IQ below 70, that is, two standard deviations below the mean. This line could have easily been drawn at one standard deviation, at an IQ 85. This is why it seems, for example, astonishingly morally arbitrary that someone with an IQ of 72 might be executed for murder, but would have been spared if their IQ was 69.^8^

The moral insignificance of biological normality and abnormality seems so obvious on reflection, is so widely accepted, and has been vigorously and, to our mind, conclusively argued, that we will simply assume it here.^9^ So we shall take it for granted that it is futile to appeal to the inherent moral significance of human nature to ground the intuitive distinction between (1) and (2).

We thus face a puzzle about the notion of harm.^10^

One way to respond to this puzzle is to conclude that, once we stop ascribing normative significance to biological normality, there is *no* interesting moral difference between (1) and (2).

This line can take two forms. One option is to conclude that *both* sets of items represent serious harms. Such a revisionary view is perhaps suggested by the arguments of some proponents of radical human enhancement. These proponents of enhancement deny, not only the moral significance of biological normality, but also the related distinction between therapy and enhancement.^1^ And to deny that curing disease is morally different from generally promoting wellbeing already seems to push us in the direction of the view that we have as much reason to increase someone's IQ from 100 to 130 as we have reason to increase it from 70 to 100 (assuming that both lead to a roughly equal increase in wellbeing), and similarly for other items on the list. Indeed, some proponents of enhancement even argue that we should divert considerable medical resources to launch a ‘war against aging’.^2^

The other option is to conclude that *neither* set of items amounts to genuine harm. This contrary revisionary view is suggested by the arguments of proponents of the Social Model of disability, who claim that conditions such as blindness, paraplegia and even severe cognitive impairment are merely differences, not genuine harms, and that, to the extent that they are even disadvantages of any kind, this is only because of the contingent environment we inhabit, and more specifically, because of current social arrangements and prejudices.^3^ On this view, many of the items on (1) are disadvantageous only in the sense that racism, sexism and other forms of social prejudice are disadvantageous. On this view, blindness can make one's life go worse (if at all) only because of current arrangements, and common prejudices against deviation from normality; once we overcome these prejudices, and see that biological normality has no moral significance, we will no longer see blindness and similar conditions as harmful.

We do not have space here to fully examine the arguments of these proponents of radical enhancement or the social model of disability.^4^ Our starting point is a widely held, intuitive normative distinction, and the common denial of the intrinsic moral significance of biological normality. Just as our puzzle does not arise for those who think that biological normality *is* morally important, it of course does not arise for those who reject the intuitive distinction. Those who reject our premises will naturally find our argument of limited interest. But these premises are very widely held.

We think, however, that even the fiercest disability advocates and most radical of transhumanists would find it hard to completely reject the intuitive distinction, though they might wish to replace some of the items on our list of examples. We doubt that many disability advocates would deny that dying very young is a serious harm, and few proponents of radical enhancement would agree that lacking telepathic powers is as much of a misfortune as being severely cognitively impaired. And nobody could plausibly hold that *nothing* is harmful or disadvantageous. But for whatever list of genuine harms one proposes, however revisionary, we believe that a similar puzzle arises.

In any event, very few of the rest of us would willingly endorse the implausible conclusion that we should treat loss of sight and lack of extraordinary longevity — let alone lack of some fantastic superhuman power — on a par.

This impasse might push some back in the direction of biological normality.^5^ If the only way to make sense of some basic normative intuitions is to accept the moral significance of biological normality then, for some, this would be a price we have to pay. It is hard, however, to see how this *could* be a price we have to pay — that it could be reasonable to return to a discredited conception of human nature just because of this moral discomfort.

If one rejects these possible responses, then it might seem that the only option left is to find some way to draw a defensible normative distinction between (1) and (2) *without* making any appeal to normality. In the next section, we will consider what we take to be the most promising ways to achieve that. We shall argue that they all fail. There is no way to draw this distinction without appealing to normality.

Where does this leave us? There is a fourth option that is easy to overlook. We can agree that, once we reject the moral significance of biological normality, there is no *deep intrinsic* normative difference between the items on (1) and (2), yet still hold that there are nevertheless morally important differences between the two lists, differences that explain the intuitive distinction that many insist on drawing — and which largely vindicate it. The aim of this article is to defend this fourth option, thus explaining away any residual attractions of the appeal to biological normality. Normality does matter, but not in the way many assume. In fact, we will show that the moral difference between (1) and (2) revolves on several surprising ways in which *statistical* normality, while lacking inherent moral significance, can nevertheless matter *derivatively*.

## 2. Can We Make Sense of Harm And Disadvantage Without Appealing to Normality?

The distinction between (1) and (2) certainly *seems* to reflect something to do with normality. Could we draw it without appealing to normality? It might be suggested that whereas we think we have a clear enough idea of how, say, blindness can reduce wellbeing, we have at best only a fuzzy sense of how high IQ or artistic talent (let alone telepathic powers) might increase wellbeing, and some would doubt whether they increase it at all. This, however, is merely an epistemic point. We can stipulate, for our purposes, that enjoyment of these conditions would significantly increase wellbeing, and that they would do so to roughly the same extent that the conditions listed in (1) decrease it. In any case, it's not especially important which items are listed in (2), since even if particular examples can be contested, it cannot be seriously denied that there are numerous conditions, capacities and abilities which, if we had had them, would have made our lives significantly better. Yet even if we make this stipulation about the effect that the lack of the conditions in (2) make on our wellbeing, it will still seem to many that there is a fundamental difference between the two lists.

Another way one might be tempted to draw the distinction is by arguing that the items on (2) merely deprive people of some possible benefit, whereas the items on (1) are *positively* harmful. But this is not plausible. For arguably, it is *not* intrinsically bad to be blind; being blind merely deprives one of access to various goods, and even that only given a certain natural and social environment. Not being able to see and not being able to read minds are in this sense on a par. Blindness is only instrumentally bad; but lacking telepathic powers is instrumentally bad in exactly the same way.^6^

It could be replied to this that blindness is an *actual* condition, the result of some actual biological process that led to an existing visual system not to function. But not having telepathic powers is merely the absence of some hypothetical (and fantastic) ability. Perhaps this distinction can draw a line with some normative weight (though we doubt this). But it is not a plausible explanation of the difference between the lists. Someone might have extremely limited intellectual capacities because of some disease; but this might also be merely a point on the normal curve, a simple reflection of their genetics, without being the product of any disease.^7^ Both ways of having extremely low IQ seem equally disadvantageous. So not all items on (1) need to be due to some dysfunction or disease. Conversely, the biological processes that prevent someone from living beyond, say, the age of ninety, are very much an actual, active condition. So this suggestion cannot draw the desired distinction.

Can we instead appeal to whether some alternative condition is *realistic* or *probable* enough? Having telepathic powers might be no more than a logical possibility, a possibility of infinitesimally small probability, and some would think that we can therefore rule it out as simply irrelevant. By contrast, having sight and being able to walk are not mere logical possibilities. But although it is indeed more probable that children will be born seeing rather than blind, this is only true at the population level. In some cases, it might be absolutely certain that some particular child will be born blind or deaf, given the genetics of its parents. And unless a cure is available, it would be utterly improbable that this person would (or could) ever see or hear. Even if, in some impersonal sense, you might say it was bad luck that someone blind was born, you cannot say that it was bad luck for *that* particular person to have been born, and remain, blind.^8^ Indeed, if probability is what makes the difference, then it seems to absurdly follow that it is more of a misfortune for someone not to win the lottery than it is for some people to be congenitally blind or have Down's syndrome. Thus even if we want to simply exclude merely fantastic capacities and possibilities from consideration, the appeal to what is realistic or probable doesn't help draw the desired distinction.

A somewhat more plausible suggestion is that the items on (1) decrease wellbeing to an especially low degree, perhaps even making some lives positively bad *on balance*, whereas the items on (2) merely lower wellbeing to some extent without preventing lives from being good enough. One problem with this proposal is that it's not especially clear how to draw a distinction between good and bad lives, as opposed to better and worse ones. It is controversial whether and how to draw a line between those lives that are worth living and those that are not — but it's at least clear that the latter must contain extreme, unremitting suffering that can't be relieved. But how are we to draw the line between a life that is good and a life that is positively bad, even if worth living? Since it's unclear and controversial where and how to even begin to draw this line, it's very doubtful that it underlies the common and intuitive distinction between (1) and (2).^9^

Moreover, while it is certainly plausible (if controversial in some circles) that someone with severe intellectual disability is likely to have significantly lesser wellbeing than someone with 100 IQ, who in turn is likely to have less wellbeing than someone with 130 IQ, this is far less plausible in the case of, say, blindness. A blind person who is healthy and wealthy, intelligent and with a sunny disposition, is very likely to have a life that is *overall* better than that of many seeing people. So, like the previous attempts to draw the distinction, this is also a false trail.

## 3. Drawing the Distinction: How Statistical Normality Can Matter

We have considered, and rejected, a range of attempts to draw a distinction between (1) and (2) without appealing to normality. This suggests to us that the initial appearance was correct: this distinction only makes sense by reference to some standard of normality. But since neither biological nor statistical normality have any intrinsic moral significance, does this force us to conclude that there is no moral distinction between the two lists? This doesn't follow. To see this, it might be useful to first step back and ask what we need the concept of harm for. We will suggest that we need the concept of harm for both explanatory and predictive purposes (its theoretical use), and to mark certain kinds of reasons for action and attitude (its normative use). And we shall argue that reflection on these uses can clarify the source of the intuitive difference between (1) and (2) — and also largely vindicate it.

### 3.1. Explanation and Prediction

Let us start with the theoretical point of the notion of harm. We often want to know *why* someone has a certain amount of wellbeing, or why he now has less wellbeing than before, or less wellbeing than someone else who is similar in various respects. Citing harm and disadvantage can help answer such questions: we can point out the fact that the person had been born blind, or became blind in an accident. We also cite such factors when we try to *predict* how something might affect people's wellbeing. We can predict, for example, that the life of a blind person is likely to have somewhat lesser wellbeing (or at least more limited opportunities for wellbeing) than the life of someone with otherwise comparable background and talents.

When we predict in this way how various factors might affect wellbeing, or why someone *in fact* has a certain degree of wellbeing, we are giving *causal explanations*. But although these are explanations of facts that *have* moral significance (i.e. in relation to someone's degree of wellbeing), such explanations are not themselves normative. They answer what are at heart *empirical* questions — questions about how and why certain things came about

Now, it is well known that causal explanations are *context-relative* — they are informative *against* some background set of assumptions. For example, in most cases the lighting of a match is taken to explain why a fire started, and the necessary presence of oxygen is taken as merely a background condition. But in some laboratory situation, the presence of oxygen might count as truly explanatory. In this way, explanations presuppose some standard of *normal* background conditions.^20^

This is also true of explanations of wellbeing. That someone does not have telepathic powers, or the intelligence of a genius, is not in ordinary circumstance relevant to explaining their level of wellbeing, and are rarely if ever a relevant comparison class. By contrast, that someone cannot see is of genuine explanatory relevance in the world we inhabit. That someone is blind will informatively explain why that person might have less wellbeing than many seeing people; that someone is not telepathic won't.

So here is one way in which the items on (1) differ from the items on (2): in typical contexts, the items on (1) can explain levels of wellbeing, relative to a set of background assumptions largely shaped by what is *statistically normal*; that we do not possess the items on (2) is usually taken for granted *in* those background conditions, and this is why these items are not usually relevant for informative explanations of wellbeing.^1^

Two points are worth noting here. First, this is importantly different from the suggestion, rejected earlier, that the distinction is due to the improbability of the possibilities ‘prevented’ by the items on (2). As we noted when we considered this proposal, the alternatives to the conditions listed in (1) can also be highly improbable, even impossible for the harmed person herself to avoid — think again of congenital blindness or Down's syndrome. But *even* when this is the case, the conditions in (1) can still offer informative *explanations* of why the harmed person has a certain level of wellbeing.^2^

Second, since explanation is context-relative, there *will* be contexts when the background assumptions are such that the items on (2) can help explain someone's level of wellbeing. If a disease makes a great painter colour blind, this *can* be a devastating misfortune, even if in most circumstances this is at most a negligible disadvantage. Similarly, an adequate explanation of how well the life of a brilliant mathematician went might need to mention that he spent much of it in futile pursuit of an important proof that was beyond his powers, and that he would have managed to achieve if he had just lived a few years longer and taken advantage of a newly available cognitive enhancer that would have increased his IQ even further beyond the norm.

### 3.2. Reasons for Action

This first way of drawing the distinction doesn't draw any *normative* distinction between (1) and (2). It only says that in most current contexts, the items on (2) have no explanatorily relevance. It does not say that the items on (1) are in any way worse, or more important. But intuitively, there *does* seem to be an important normative difference between the two lists. So this could not be the whole story.

Harm and disadvantage are not only explanatory notions. They also typically have normative significance, most familiarly by generating various *reasons for action*. The question, then, is whether the items on (1) and (2) generate different kinds of reasons.

Now, when we can remove some obstacle to greater wellbeing, then we have at least a *pro tanto* reason to do so, and in many cases it would be morally wrong to fail to remove that obstacle, let alone to intentionally impose it. If blindness reduces wellbeing, and we can cure it, we should. If higher IQ increases wellbeing, and we can raise it, we should, other things being equal. More controversially, it can be argued that we also have reasons to create children with characteristics that are likely to lead to greater wellbeing, rather than other possible children with a lesser potential for wellbeing.^3^ If we can choose, we should choose to create seeing, highly intelligent children rather than ones who are blind or of lower intelligence. These points apply to items on *both* (1) and (2).

This is what we meant when we said that there is no *deep* normative distinction between (1) and (2). The items on both lists are obstacles to greater wellbeing, and they potentially generate the same kind of reasons for action, even though, of course, there are some such obstacles that we are not now, and probably never will be, able to remove.

There is no deep, inherent moral distinction between the two lists. But it *doesn't* follow that it simply doesn't matter, or isn't important. Here is why. Limitations of resources mean that we can rarely promote wellbeing in all possible ways. We have to choose. So the question is whether we give priority to curing paraplegia or blindness than to increasing intelligence or artistic talent, even if — let us assume for argument's sake — both lead to broadly the same increase in wellbeing.

This is a question about distributive justice, and the answer one gives naturally depends on one's view of justice. We obviously do not have the space here to defend any particular conception of justice. But we can draw attention to the implications of intuitively plausible and influential conceptions of justice.

On one influential view of distributive justice, we should give priority to the worst off.^4^ It is arguable — though, we emphasize, not necessarily always true — that the wellbeing of someone with paraplegia is often lower than the wellbeing of someone with merely average intelligence. To the extent that items on (1) tend to make people's lives significantly worse than the lives of *most* others, considerations of justice might give priority to the prevention or correction of these conditions. This priority would again often reflect statistical normality: after all the worst off are exactly the people who are located at the further end of the bell curve of wellbeing in some population.

However, as we saw earlier, it is not at all clear that *all* the items that intuitively belong in the list in (1) significantly reduce wellbeing, even when we consider, as is appropriate in matters of policy, broad trends in the population rather than individual cases. Still, on some views of justice we might have reason to give priority to people who suffer from these conditions even if they do not lead to overall worse lives than the norm. To the extent that we value equality of *opportunity*, then the relative rarity of the items on (1) would again give us reason to give them priority. If most people have average IQ, then the opportunities of people with average IQ are similar to those of most other people. But far fewer actual people are blind, and these people therefore lack opportunities available to most others. This could again give reasons to give priority to curing blindness than to raising normal IQ. So, again, statistical normality can make a moral difference in virtue of how its bears on considerations of justice.^5^^,^^6^

We have just considered how considerations of justice can give priority to the prevention or cure of some conditions compared to others. In some cases, such a cure is simply not available. There might be no way to cure someone's blindness, and it is at least conceivable that in some cases, a person's condition — an example might be Down's syndrome — is constitutive of their biographical identity so that even if a cure was possible, it would result in what is, in the relevant sense, a different person.^7^ Even in such cases, there might be reasons to change the environment (e.g. by providing appropriate aids, facilities, support, etc.) to counteract the negative effects the condition would otherwise have. When it is impossible or simply not feasible to change the environment in this way, justice might still require that we *compensate* persons for their condition. The considerations about priority outlined above would also apply to changing the environment and to compensation. Because justice requires us to give priority to the worse off, we are likely to have reason to give far greater priority to helping and compensating blind people than to helping people of merely average IQ.^8^

This, then, is the second way of justifying the distinction between (1) and (2). Although this distinction doesn't reflect a deep moral division, the impression that the items in (1) are in some way worse, or more urgent, is not illusory, since considerations of justice can lead us to give significant moral priority to the items on (1). Although normality does not matter in itself, it is tied in important ways to the distribution of abilities and conditions in a population, and can thus, by impacting on justice, give greater priority to certain reasons for action.

### 3.3. Reasons for Attitudes

Harm and disadvantage generate not only reasons for action, but also reasons for attitudes such as regret and sympathy. Because of the practical focus of much of ethics, these reasons are often neglected. But as we saw, one source of the impression of intuitive difference between (1) and (2) is that only the items on (1) seem to merit our sympathy and regret — that it seems absurd to feel regret that someone lives ‘only’ to the age of 100. And this seems to suggest that the items on (1) are in some distinctive way *worse*. We shall now argue that this impression can also be explained (and largely vindicated) by reference to the derivative significance of statistical normality.

When we cannot improve some adverse condition, we still have reason to regret it. Regret can come in both personal and impersonal forms. I can regret, *for* some person's sake, the fact that *this* person was born blind. Or I can impersonally regret the fact that *some* people are born blind. This is not an idle distinction: if, for some person, it *couldn't* have been the case that they had not been born blind, it makes no sense for them to regret this or for us to regret it for their *own* sake. We can only regret this in an impersonal sense.

Now it would not be incoherent in the same way for us to regret, for the blind person's sake, the fact that they *remain* blind. But regret does seem *irrational*, or at least pointless, when a cure for the person's blindness is highly improbable or even merely a distant logical possibility — just as it seems irrational to regret that one isn't a greater author than Shakespeare. This suggests that our attitudes should respond not only to the final or instrumental value of some possibility, but also to its probability.^9^

We have already considered the suggestion that the difference between (1) and (2) reflects the improbability of the alternatives listed in (2). And we saw that this suggestion fails: it is at least as improbable that someone with congenital blindness or Down's syndrome would be born normal. This means that, so long as our attitudes are personal in character, then we should have different attitudes to the blindness of people who were born blind as an inevitable result of their genetic endowment, and to that of people who became blind through a preventable disease. We should similarly have different attitudes to blindness when some prevention or cure is available or at least vaguely probable, and to blindness when no cure is remotely in the offing. It is thus *not* the case that the items on (1) *always* deserve special sympathy or regret, or even any sympathy at all. In this particular context, the intuitive distinction between (1) and (2) is indeed mistaken.

But there is a more limited way in which the items on (1) and (2) might still call for different attitudes. The idea is similar in form to the normative distinction we drew above concerning reasons for action. Besides degree of badness and probability, our attitudes should also be sensitive to comparative factors — our attitudes are a limited resource, and the way we should ‘distribute’ this scarce resources echoes considerations of distributive justice. And this means that if most people have a certain level of intelligence, then having lesser opportunities because one has intelligence far below the normal deserves more of our attention than not being super-intelligent when most people have average intelligence. This is so even if this lack leads to the loss of the same amount of good, and even if the alternative is *equally probable*. Thus, given the *actual* normal distribution of abilities in our society, a cognitively impaired person can deserve more of our sympathy than someone of merely average intelligence. This is the third way in which statistical normality helps explain the intuitive difference between (1) and (2).

## 4. Other Ways in Which Normality Can Matter

It is worth quickly mentioning two weaker ways in which normality might make a normative difference that broadly corresponds to the apparent distinction between (1) and (2).

First, many people are consciously or unconsciously concerned about their status — about how they fare relative to other people. To be located below the common standards of normality can be a source of frustration and unhappiness for at least some people. This frustration and unhappiness can also be generated and reinforced by the negative attitudes that the normal sometimes unfortunately exhibit towards the abnormal — and these pernicious attitudes are of course also a separate source of harm. This might be a further reason why the items on (1) *seem* worse than those on (2) — though considerations of status aren't likely to give much *support* to the intuitive distinction.

Second, although the statistically and biologically normal has no *inherent* moral significance, they can matter *to* us if we have formed deep attachments, often sustained over a long history, to common features of *Homo sapiens*, or even more locally, to the statistically normal features of human beings in our own society. Such attachments can generate reasons to be *partial* to these features in the roughly same way that deep personal attachments can generate reasons to be partial towards our family and friends. We may, in this way, care more about sight than about imaginary telepathic powers, or less about super-intelligence than about common human intelligence.^30^ But such partial reasons seem to us rather weak. They most certainly cannot justify giving preferential treatment to the normal, or defeat any but the weakest reasons to promote wellbeing in ways that go beyond the normal; to our mind, they might at most make it justifiable for some people to wish to stay as they are when it becomes possible to remove some of the obstacles to wellbeing on lists like (2) — for example, to refuse to undergo biomedical enhancements that would dramatically increase their lifespan. But it is at least questionable whether it would be rationally defensible for parents to refuse such enhancements for their children on these grounds. In any case, while it's easy to see how many people would ascribe such partial value to the human form, or to some core psychological features of humans, it is harder to see what would lead people to form deep attachments to the current average levels of human IQ or lifespan.

## 5. Conclusion

The lives of persons go badly or well to various degrees. They do so by containing more or less wellbeing — more or less of what is intrinsically good or bad for us. Various causal factors play a role in explaining why different lives possess the degree of wellbeing that they do. These factors are not (or need not be) intrinsically good or bad; they may be of only instrumental value; they possess value only because of their effects.

The notions of harm and disadvantage are often used to refer to conditions that are instrumentally bad in this way. These notions are themselves instruments — that is, they serve various useful functions, helping us make sense of the wellbeing people have or are expected to have. They guide our acts and shape our attitudes.

Since a range of factors can influence what counts as an informative explanation, and what reasons we have to act or feel with respect to some condition, it is hard to define these notions very precisely. Many have been tempted to understand these notions in terms of deviations from normal species functioning — from what is biologically normal. With many others, we believe that biological normality cannot serve as such a moral foundation. An attractive alternative instead grounds these notions in that of wellbeing: the harmful and disadvantageous is what adversely affects wellbeing.^1^ But this can seem to lead to highly counterintuitive results, pushing us back in the direction to the discredited foundation of biological normality.

This retreat would be a mistake. We do not need to appeal to biological normality, and its alleged moral significance, to explain — and largely vindicate — the intuitive distinction between different ways in which lives could have been better. What grounds this distinction isn't biological normality, but *statistical* normality, and the ways in which it can have a derivative normative importance. Statistical normality can bear on what counts as a good explanation, on what we should feel, and, when coupled with plausible conceptions of justice, on our reasons for action. Since it's utterly implausible that statistical normality matters in itself, and because statistical normality is often also *correlated* with biological normality, this normative pattern can give the mistaken impression that biological normality matters, or underlies some deep moral distinction. We predict, however, that as biomedical advances transform human capacities in the future, and statistical normality and biological normality drift further apart, this impression will dissolve.

Future developments are likely to change the way we understand harm in a further way. When societies empower large numbers of people with new powers through technology, it can become harmful and unjust to lack the abilities provided by technology. To lack a computer or a mobile phone is, in the Western world, to be disadvantaged. Justice might require the provision of such technology as a means to promoting wellbeing ahead of some medical treatments for minor diseases, such as benign fungal toe-nail infections. It seems to us likely that in the near future, it will be recognized that the items on list (1) should include not only biological deviations from normality but also technological deprivations.^2^

Our intuitions about harm and disadvantage can suggest that biological normality has deep moral significance. We have argued that this impression is mistaken, and can be explained away. What really matters is wellbeing, how it is justly distributed, and what affects it.^3^

## Notes

1 Disadvantage and harm are overlapping though not perfectly synonymous notions. We will largely use them interchangeably, as they both fall within our target normative phenomenon. The items we list are examples of things widely held to be serious harms, but we recognize that some will strongly disagree. To an important extent, this disagreement doesn't affect our argument, since those who think that, say, blindness isn't a genuine harm can simply substitute it with something they *do* find harmful. But we will later briefly address such critiques of common views about disability. We have already discussed such critiques, and explain how such conditions can cause harm and constitute disability, in J. Savulescu & G. Kahane, ‘The moral obligation to create children with the best chance of the best life’, *Bioethics* 23 (2009): 274–290; G. Kahane & J. Savulescu, ‘The welfarist account of disability’ in K. Brownlee & A. Cureton (eds) *Disability and Disadvantage* (Oxford: Oxford University Press, 2009), pp. 14–53; J. Savulescu & G. Kahane, ‘Disability: A welfarist approach’, *Journal of Clinical Ethics* 6 (2011): 45–51.

2 There is no agreed consensus about the nature of wellbeing. Hedonist theories identify it with having being happy and not suffering. Desire fulfilment theories identify it with having our desires fulfilled. Objective theories claim that certain states and activities are objectively good — these can include pleasure, but also deep personal relationships, knowledge, development of talents, virtue, and so forth. For discussion, see J. Griffin, *Wellbeing* (Oxford: Oxford University Press, 1986). To a large extent, this disagreement doesn't bear on our argument here since these theories nevertheless largely agree about substantive questions about wellbeing (e.g. close personal relationships can make us happier, or satisfy common desires.) For one point where this disagreement might make a difference, see Endnote 5.

3 This is a widely held understanding of harm. Joel Feinberg, for example, claims that ‘for A to harm B, he must cause B's personal interest to be in a worse condition than it would have been in had A acted differently’: Joel Feinberg, ‘Wrongful life and the counterfactual element in harming’, *Social Philosophy & Policy* 4 (1987): 145–178.

4 To repeat: we use blindness just as an example of something widely held to be disadvantageous. Those who deny this can consider instead some other condition they *do* think is seriously disadvantageous. Still, some remarks are in order. Denials of the disadvantage of blindness and other impairments often appeal to the finding that individuals who have serious impairments report levels of subjective wellbeing no different from those of healthy or ‘normal’ individuals (for an illuminating discussion, see H. Carel, ‘“I am well, apart from the fact that I have cancer”: Explaining wellbeing within illness’ in L. Bortolloti (ed.) *Philosophy and Happiness* (Basingstoke: Palgrave Macmillan, 2009)). In thinking about this striking finding, we should first bear in mind that this phenomenon of ‘hedonic adaptation’ extends very far. It even encompasses, for example, the majority of individuals who suffer from ‘locked-in syndrome’ — individuals whose only contact with the world is through blinking (Bruno et al., ‘A survey on self-assessed wellbeing in a cohort of chronic locked-in syndrome patients: Happy majority, miserable minority’, *British Medical Journal Open* (2011): doi:10.1136/bmjopen-2010-000039). And as Sen has pointed out, even the subjugated and oppressed often adapt their preferences to their unfortunate circumstances, and can be satisfied with them: A. K. Sen, *The Standard of Living* (Cambridge: Cambridge University Press, 1985). Second, to the extent that these studies really show that these conditions *don't* reduce wellbeing in *any* respect, they would have dramatic revisionary implications. They could imply that there is *no* significant reason to regret the fact that someone became, say, locked-in, and that to make someone blind *isn't* harmful or especially wrong. And even if many blind individuals feel quite happy, it's safe to presume that they would still greatly fear becoming completely paralyzed — and vice versa. So we believe that only few, if any, would endorse this revisionary view in an unqualified way, once its implications are fully spelled out. Third, we *do* agree, however, that this research suggests that many conditions are not *as* bad as many assume. This is important. But we believe that wellbeing also has objective dimensions, and it's harder to defend the claim that these aren't at all affected by, e.g., blindness or paraplegia. Still, how such conditions affect wellbeing is a difficult empirical and normative question that cannot be answered simply by appealing to any commonsense consensus.

5 Again, if this list seems contentious, pick some talent or capacity that you believe does make a life go especially well, such as having a superb sense of humour, or great originality, or an exceptional ability to accurately interpret and respond to other people's emotions, etc.

6 See e.g. Philippa Foot, *Natural Goodness* (New York: Oxford University Press, 2001).

7 Notice that we needn't deny here that biology can draw some principled naturalistic line between the items on (1) and (2). For example, something like Christopher Boorse's influential naturalist account of disease might be thought to do the job (cf. his ‘On the distinction between disease and illness,’ *Philosophy and Public Affairs* 5 (1975): 49–68) — though we doubt this. The point we are making is that such a biological distinction still won't help ground the intuitive *normative* distinction between the two lists. Notice also that by ‘normative’, we refer to moral and prudential norms. Some authors think that we can ascribe biological functions to organisms and their parts, and these functions can be thought to set biological norms of assessment. But, again, the point is that norms in this thinner sense do not in themselves generate any *moral* or *prudential* reasons of any kind. We discuss the normative failure of naturalistic accounts of disability in Kahane & Savulescu 2009 op. cit.

8 A woman called Teresa Lewis was recently executed in Virginia because she was found guilty of conspiring to murder and her IQ was 72 (Ewen MacAskill, ‘Teresa Lewis given lethal injections despite protests over low IQ’, *The Guardian* 24 September 2010. Online at: http://www.guardian.co.uk/world/2010/sep/24/teresa-lewis-lethal-injection-virginia [accessed 9 June 2011.]) If you have intellectual disability in Virginia, you can't be executed. Yet there would have been no significant functional difference between a person with an IQ of 69 and 72. Indeed, 69 IQ is probably within the error margins of IQ tests. Of course any *sharp* distinction here would be somewhat arbitrary. Our point is rather that this precise line is *tragically absurd*, because the *overall* line is, in this context, morally arbitrary — it is not based on any serious attempt to identify a threshold level of mental capacities required for moral (and thus legal) responsibility.

9 For just a few examples, see Philip Kitcher, ‘Essence and perfection’, *Ethics* 110 (1999): 59–83; Jeff McMahan, *The Ethics of Killing: Problems at the Margins of Life* (New York: Oxford University Press, 2002), pp. 209–228, and his ‘Our fellow creatures’, *Journal of Ethics* 9 (2005): 353–380; Tim Lewens, ‘Foot note’, *Analysis* 70 (2010): 468–473; Allen Buchanan, *Beyond Humanity* (Oxford: Oxford University Press, 2010). The biologist Michael Ghiselin goes even further. He writes: ‘What does evolution teach us about human nature? It teaches us that human nature is a superstition’: Michael Ghiselin, *Metaphysics and the Origin of Species*, (Albany, NY: SUNY Press, 1997), p. 1.

10 This is a challenge, not only to accounts of harm and disadvantage, but also to some accounts of disability. See Kahane & Savulescu 2009 op. cit. Similar problems arise in other contexts. See e.g. Leo Katz, ‘What to compensate? Some surprisingly unappreciated reasons why the problem is so hard’, *San Diego Law Review* 40 (2003): 1347–1353 and the discussion of the badness of death in McMahan op. cit., chapter 2, esp. pp. 98–117. McMahan's remarks on misfortune relate to our topic here, but we don't have the space to draw the connections.

20 See e.g. John L. Mackie, *The Cement of the Universe*, (Oxford: Oxford University Press, 1974).

30 We draw here on G. A. Cohen, ‘Rescuing conservatism: A defence of existing value’, in R. J. Wallace, R. Kumar, and S. Freeman (eds) *Reasons and Recognition: Essays on the Philosophy of T. M. Scanlon* (Oxford: Oxford University Press, 2011). See also G. Kahane and J. Savulescu, ‘The value of sex in procreative reasons’, *American Journal of Bioethics* 10 (2010): 22–24.

